# Missing Microbes: How the Overuse of Antibiotics Is Fueling Our Modern Plagues

**DOI:** 10.3201/eid2011.141052

**Published:** 2014-11

**Authors:** Andi L. Shane

**Affiliations:** Emory University School of Medicine, Atlanta, Georgia, USA

**Keywords:** antibiotics, antimicrobial resistance, antimicrobial drugs, microbiome, obesity, juvenile diabetes, asthma

This engaging book on the importance of the microbiome in human health weaves the personal and professional experiences of its author, Dr Martin Blaser, into a thought-provoking commentary on the perils of inappropriate antimicrobial drug use. In 16 chapters, Blaser, director of the New York University Human Microbiome Program, builds a case for recognizing the importance of commensal organisms, describes the effects of overuse of antibiotics on human ecology, and cites both personal and professional experiences to support his concerns.

Blaser proposes that perturbations to the human microbiome have led to an increasing incidence of obesity, juvenile diabetes, and asthma, which he terms “modern plagues.” He uses a discussion of his work with *Helicobacter pylori* to illustrate the concept of amphibiosis, a phenomenon in which an organism may be friend or foe, depending on the environment of the host. Blaser proposes that the eradication of *H. pylori *and the inflammatory gastritis that was associated with its presence resulted in a replacement of ulcers and gastric cancer with heartburn and esophageal adenocarcinoma. He also posits that celiac and inflammatory bowel diseases may be the consequence of alterations in bowel flora resulting from multiple courses of antimicrobial drugs. He cites the example of his daughter to support this theory, tying her clinical diagnosis of celiac disease to the multiple courses of amoxicillin she received as a child for otitis media and courses of metronidazole she received for traveler’s diarrhea during international travel while she worked in resource-challenged settings.

Blaser draws on his experiences as an infectious disease specialist, chair of medicine, advisor, and epidemiologist at the Centers for Disease Control and Prevention to compare and contrast the alterations in our microbiome with the consequences of climate change. He describes the individual and societal implications of these events and warns against an “antibiotic winter” in which antimicrobial resistance and disease prevail because of the destruction of our internal and external ecosystems.

In the final chapter and epilogue, Blaser proposes solutions that may be implemented at the individual and community levels. As initial steps, he proposes avoiding antimicrobial drugs as growth promoters in animals; minimizing the use of these drugs for unclear indications in infants, whose gut flora are still being established; and considering how each drug course will affect the individual and the community. 

Despite his emphasis on missing microbes, however, Blaser is wary of using probiotics, prebiotics, and synbiotics to restore microbial balance. He reasonably cites the challenges arising from the unsubstantiated claims of manufacturers and the paucity of well-designed trials to evaluate probiotics. What he neglects to mention is that the requirements imposed by government agencies in North America and Western Europe make probiotic studies infeasible, if not impossible, in the current regulatory climate.

Overall, this book is an important and worthwhile read for members of the general public and the scientific community. Blaser’s appealing and personable prose is captivating, easy to read, and convincing and reminds us that health care advances also have consequences.

